# Effect of the COVID-19 pandemic on contraceptive prescribing in general practice: a retrospective analysis of English prescribing data between 2019 and 2020

**DOI:** 10.1186/s40834-022-00169-w

**Published:** 2022-03-14

**Authors:** Susan H. Walker

**Affiliations:** Faculty of Health, Education, Medicine and Social Care, Anglia Ruskin University, Bishop Hall Lane, Chelmsford, CM1 1SQ UK

**Keywords:** Prescriptions, General practice, Contraception, COVID − 19, England

## Abstract

**Objectives:**

This paper looks at how contraceptive prescribing by General Practices in England was affected by the COVID-19pandemic and lockdown. It compares English prescribing data in April – June 2019, the year before COVID19, and April–June 2020, the first three months of ‘lockdown’.

**Design & Setting:**

This paper is based on retrospective analysis of the English Prescribing Dataset which reports monthly on prescribed items from English General Practices. Data on all forms of prescribed contraceptive methods were extracted using BNF codes, and total quantities tabulated by method. To reach the total number of months of contraception provided, total quantities were divided or multiplied according the frequency with which the method is taken per month or the numbers of months of contraception provided.

**Results:**

Prescription of the combined oral contraceptive pill reduced by 22% during the period of lockdown compared to the same three months in 2019. Prescriptions of Progestogen-Only pills remained stable. Prescription of long-acting methods reduced, with the greatest reductions in implants (76% reduction from pre-lockdown levels), intra-uterine systems (79% reduction from pre-lockdown levels) and intrauterine devices (76% reduction from pre-lockdown levels).

**Conclusions:**

The disruption of face-to-face contraceptive consultations in General Practice during a COVID-19 ‘lockdown’ has resulted in a reduction in oestrogen –containing methods compared to progestogen-only methods, which require less face-to-face monitoring.

Implant and intrauterine contraceptive device prescription reduced by three quarters over the first three months of lockdown, which has the potential to result in a rise in unintended pregnancies.

## Introduction

There have been concerns raised worldwide that lockdown restrictions have hampered women’s access to contraception and safe abortion, and arguments that these services should be prioritised to prevent unwanted pregnancies and pregnancy-related deaths [1] [2] [3] [4] [5].

The COVID19 pandemic caused England and the rest of the UK to enter lockdown on 23rd March 2020. Although GP surgeries remained open throughout the lockdown, patterns of working changed, with face-to-face consultations initially reduced to those considered essential [6]. Personal Protective Equipment (PPE) became more readily available, and practices adapted to working in a COVID-19 safe manner, more routine services gradually resumed, where possible remotely or virtually [7, 8]. Patients were permitted to attend medical appointments throughout the period of lockdown, but anxiety about the safety of healthcare premises, problems with caring for children who were not in school, and difficulties with public transport are likely to have made attending GP surgeries for contraceptive provision more difficult. It is estimated that about 80% of women access contraception through their GP [9]. At the same time community contraceptive clinics were experiencing similar constraints and restrictions on access.

The Faculty of Sexual and Reproductive health (FSRH) produced guidance for supplying contraceptive services during the pandemic. These identified essential services for women (see Table 1), and suggested changes that would allow routine provision to be provided online [10].

**Table 1:** Essential Sexual & Reproductive Health services during lockdown

• Emergency contraception (oral and, where possible, fitting copper intrauterine device - IUD)	
• Support existing, continued use of Long-Acting Reversible Contraception (LARC)	
• LARC complications	
• Contraception for vulnerable groups	
• Abortion care and post-abortion contraception	
• Sexual assault care	

Further guidance was issued regarding short-term measures to enable women to remain contracepted even when unable to access services face to face, including guidance on the safety of the progestogen-only-pill (POP) as a bridging method, how to manage the need for Blood Pressure (BP) and Body Mass Index (BMI) measurement for the Combined Oral contraceptive Pill (COCP) and advice on instituting and renewing Long-Acting Reversible Contraceptive (LARC) methods [11, 12].

Lack of access to face-to face consultations, and caution on behalf of healthcare professionals in regard to prescribing without examination will have caused a change in prescribing habits, and subsequent effects on the contraception used by women.

The traditional pattern of visiting a doctor or clinic to obtain contraception underwent a forced change in England as a result of the restrictions imposed by the COVID-19 pandemic. This has two implications which make it important to understand the changes in contraceptive prescribing in a defined area during this time. The first and most immediately pressing need is to recognise the potential for unintended pregnancy as a result of women becoming unable to access their usual method of contraception or needing to use a less preferred method. Women need to be enabled to use their preferred method again as soon as possible, as services return to normal. Secondly the restrictions imposed by the pandemic have enabled a form of natural experiment to take place, in which the usual pattern of face-to-face consultation, often driven by medico-legal concerns, had to adapt. This forced clinicians to find new ways of supplying progestogen-only pills remotely, and novel ways of carrying out the required blood pressure measurements for combined hormonal methods. In an age when working women may find it more convenient to obtain contraceptive supplies without needing to attend a face-to-face appointment, understanding what was and was not possible for safe prescribing should be better understood, so the beneficial aspects of these changes might be retained.

This paper aims to analyse how the COVID-19 pandemic impacted contraception prescription in England during 2020.

## Method

This paper draws on data from the English Prescribing Data (EPD) set published by the NHS Business Services Authority (https://www.nhsbsa.nhs.uk/prescription-data/prescribing-data/english-prescribing-data-epd) [13]. This database contains detailed information on prescriptions issued on a monthly basis by every General Practice in England, and dispensed in Great Britain, the Channel Islands and the Isle of Man. It excludes items not submitted for dispensing, prescriptions issued in hospitals, and prison, and private prescriptions. It also excludes any patient identifiable data so this data can show practice level variations in prescribing, not individual level use of contraceptive methods.

The dataset provides numbers and details of prescribed items (e.g. desogestrel 75 microgms) and the quantity of drug dispensed (e.g. 84 tablets). It also supplies the total quantity prescribed, derived from the number of items multiplied by the quantity (e.g. 3 × 84 tablets of desogestrel 75 mg = Total Quantity of 168). Drugs are listed by British National Formulary Chapter, Section and presentation, and by both generic drug names and tradenames.

Data for all prescriptions, from all practices in England, for April, May and June 2020, the first three months after lockdown, were compared with the same three months the previous year (April, May, June 2019). Thus the study is a longitudinal study using two time windows (April–June 2019 and April to June 2020) to examine changes in prescribing by all English GP practices during the two time periods. Data on all forms of prescribed contraceptive methods were extracted using BNF codes and descriptions, and total quantities tabulated by individual method. Data was extracted from the database for each month, using an Excel Data Query, and by searching for items by truncated BNF code (*denotes truncation). The codes used are presented in Table 2. BNF Descriptions were used to further identify and separate the individual methods, using an Excel Pivot Table, extracting for each BNF description the name and total quantity prescribed for the month in question. Items were described either generically or by tradename, according to what the prescriber requested on the prescription, so these items were not counted twice.

**Table 2:** BNF codes and descriptions used to extract data

Truncated BNF code	Contraceptive method	Examples of BNF description
070301*	Combined Hormonal Contraceptive Methods	Mercilon 150microgram/20microgram tablets
		Ethinylest 33.9microg/Norelgestromin 203microg/24 h ptch
070302*	Progesterone Only methods	Medroxyprogesterone 150 mg/1 ml inj pre-filled syringes
		Levonorgestrel 20micrograms/24 h intrauterine device
		Desogestrel 75microgram tablets
210400*	Contraceptive devices	Copper T380 A intrauterine contraceptive device
		Nova-T 380 intrauterine contraceptive device
070305*	Emergency Contraceptive pills	Levonelle 1500microgram tablets
		Ulipristal 30 mg tablets

To calculate the total number of months of contraception provided by each method from the total quantities prescribed, total quantities were divided or multiplied according the frequency with which the method is taken per month or the numbers of months of contraception provided. For example the total quantity of a 21- day COCP is divided by 21 to calculate the numbers of months of contraception provided, whereas total prescriptions for a 5 year Intra-Uterine System (IUS) were multiplied by 60 months to calculate the number of months of contraception provided.

This allowed the number of months of contraception issued by all General Practices in England during the first three months of lockdown (March–May 2020) to be calculated and compared with the corresponding months in the previous year (2019).

### Patient and public involvement

No patients were involved in the design of this study.

## Results

Short-acting pills (COCP and POP) were the methods prescribed most in both periods in terms of total quantity of items, and in terms of numbers of months of provision (Table 3).

**Table 3:** Total months of contraceptive provision by method

Total Months of supply	***CHC all***	COCP	Patch	Ring	***PO all***	POP	Injection	Implant	***IUC all***	IUS	IUD	***All EC***	LNG EHC	UPA EC
**Apr19**	*1,372,132*	1,336,916	28,894	6322	*2,388,779*	1,087,609	241,678	389,340	*1,011,432*	670,152	341,280	*9397*	6140	3257
**May19**	*1,529,103*	1,488,803	32,890	7410	*2,656,959*	1,211,044	253,187	431,568	*1,137,180*	761,160	376,020	*10,100*	6607	3493
**Jun19**	*1,429,162*	1,390,976	31,541	6645	*2,500,590*	1,112,190	235,464	412,128	*1,106,448*	740,808	365,640	*9650*	6388	3262
**Apr20**	*1,167,013*	1,128,523.67	31,889	6147	*1,585,174*	1,191,055	193,323	74,880	*196,416*	125,916	70,500	*5429*	3660	1769
**May20**	*1,075,833*	1,040,898	29,394	5541	*1,427,642*	1,063,629	193,961	67,284	*167,628*	102,768	64,860	*6630*	4369	2261
**Jun20**	*1,165,342*	1,126,192	32,939	6211	*1,724,337*	1,120,560	208,773	157,680	*366,384*	237,324	129,060	*8821*	5803	3018

*CHC* Combined Hormonal Contraception, *COCP* Combined Oral contraceptive Pill, *PO* progesterone only methods, *POP* progesterone Only Pill, *IUC* intrauterine Contraception, *IUS* intrauterine system, *IUD* Intrauterine Device, *EC* Emergency contraception, *LNG* Levonorgestral emergency Hormonal contraception, *UPA EC* Ulipristal acetate emergency contraception

Due to their longer action, Long-Acting Reversible method (LARCs) are represented in greater amounts in terms of number of months of provision despite fewer prescriptions being issued.

The total number of months of prescribed contraceptive provision, excluding emergency contraception (EC) in April 19 was 4,102,191 months, in May 19 was 4,562,081 months and June 19 was 19, 4,295,391 months. This reduced to 69% (2,822,687 months) of April 19’s provision in April 20, and to 56% (2,568,335 months) of May 19’s provision in May 20, before recovering slightly to 70% (3,018,739 months) of June 19’s provision in June 20.

Combined Oral Contraceptive pills (COCP) and Progestogen-Only pills (POP) accounted for the bulk of Progestogen Only (PO) and combined hormonal (CHC) prescribing in both time periods.

**Fig. 1:**
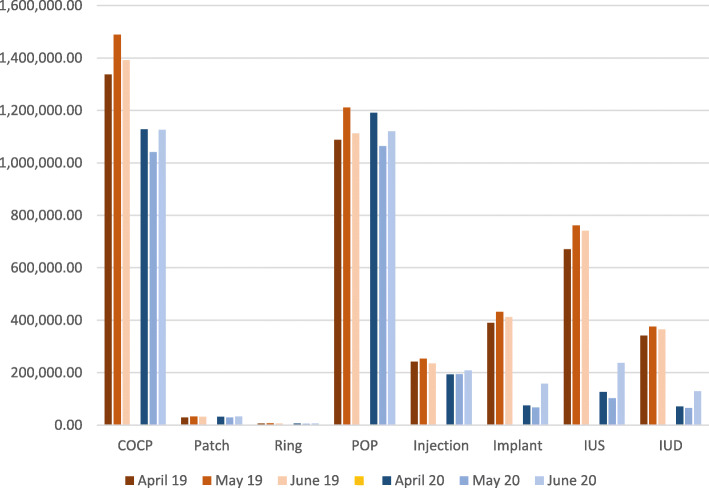
All methods by Months of Contraceptive Provision

There was a 21% decrease in the prescription of oestrogen-containing CHC methods, in terms of months of provision between April–June19 (4,330,397 months) and April 20–June20 (3,408,188 months). There was a 37% reduction of Progestogen Only method prescription between the April–June 19 (7,546,327 months) and April–June 20 (4,737,154 months) (Fig. 1).

The reduction in CHC prescription was almost entirely due to reduced prescription of COCP. COCP prescription reduced by 22% from April–June 19 (4,216,694 months) to April to June 20 (3,295,614 months).

Comparing the ratio of POP to COCP provision, the total number of months of provision by COCP over April, May and June 19 was 24% higher (4,216,694 months) than that provided by the POP (3,410,842) prior to lockdown, but almost equivalent during lockdown (3,295,614 months COCP v. 3,375,245 months POP).

In contrast to CHC prescription, the reduction in PO provision was due to a marked reduction in prescriptions of long-acting methods, i.e. implants (75% reduction; 1,233,036 months v. 299, 844 months) and intrauterine systems (79% reduction; 2,172,120 months v. 466,008 months) between April–June 19 and April – June 20.

The prescription of the POP remained at 99% of its pre-lockdown level in April–June 2020.

**Fig. 2:**
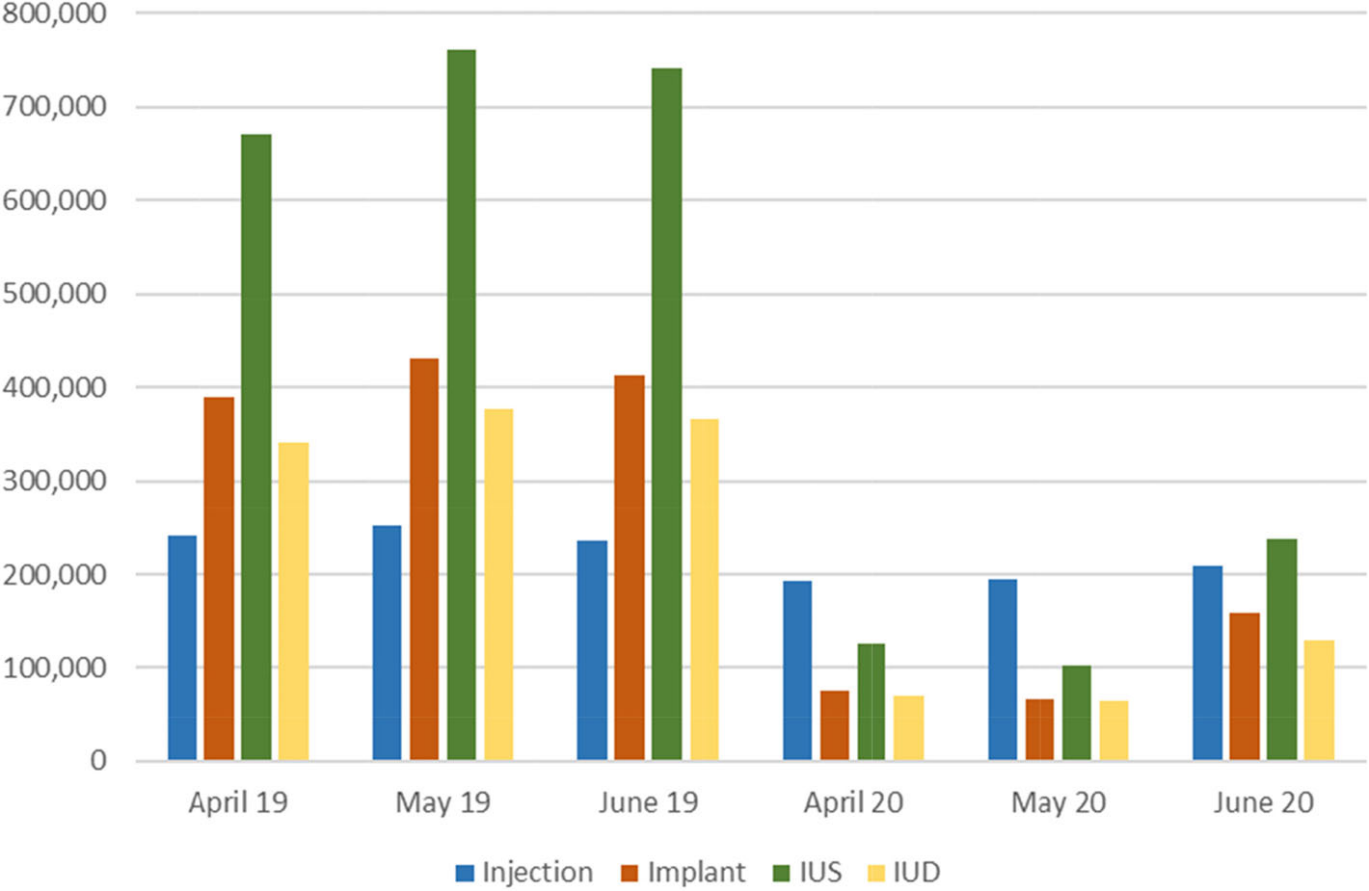
LARCs by Months of Contraceptive Provision

Of the Long-Acting Reversible Contraceptive (LARC) methods (Implant, injection, Intrauterine System and Intrauterine Device) the Intrauterine System (IUS) provided most months of contraception prior to lockdown, but this reduced during lockdown, when the contraceptive injection was the method providing most months of provision between April and June 20 (Fig. 2).

Amidst the overall reduction of injection provision there was a small change from intra-muscular (IM) to sub-cutaneous (SC) contraceptive injection provision, which can be self-administered by women. In April–June 19 only 8% (18,735) of contraceptive injections were sub-cutaneous compared to 15% (25,987) in April–June 20.

Total Intra-uterine contraceptive provision fell by 78% from 46,969 prescribed items (3,255,060 months of provision) in April–June 19 to 10,393 items (730,428 months of provision) in April–June 20.

**Fig. 3:**
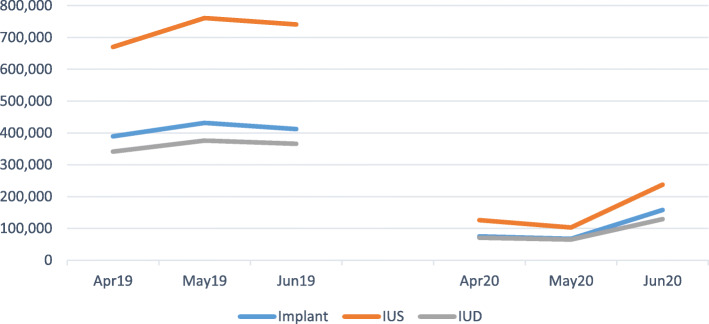
Implants, IUD & IUS by Months of Contraceptive Provision

As General Practice re-organised in response to lockdown provision of implants, and intrauterine contraception began to recover from a low point in May 20. However months of contraception provided by implants were 24% of pre-lockdown levels in April–June 20, IUS 21% of pre-lockdown levels, and copper Intra-uterine Device (IUD) at 24% of pre-lockdown levels respectively (Fig. 3).

### Provision by GPs of emergency contraception during lockdown

**Fig. 4:**
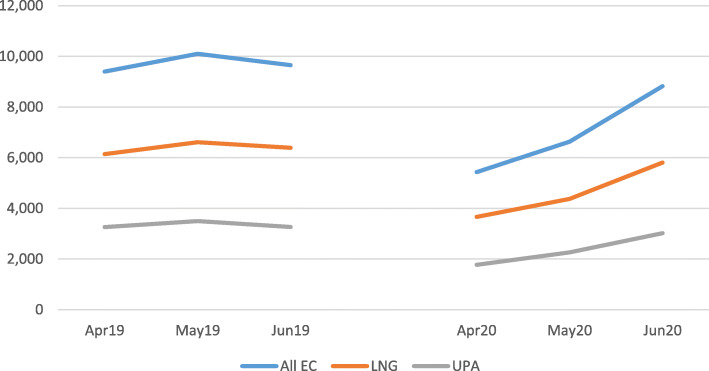
EC all – by total items prescribed

The prescription of Emergency Contraception prescription by GPs decreased by 42% between April19 (9397 items) and April 20 (5429 items) but began to increase over May and June 20, beginning to reach pre-lockdown measures in June 20 (8821 items) (Fig. 4).

## Discussion

During the period of lockdown in 2020 when face-to-face consultations were restricted or hard to attend, overall prescribing of contraception in General practice was reduced, compared to the same 3 month period in 2019.

Prescribers reduced their supply of oestrogen containing COCP, but maintained the quantity of POP prescribed, presumably in response to the safer profile of the POP in situations where blood pressure and weight could not be measured at the time of prescription. The POP may also have been provided to help women to bridge the time when their usual LARC method should have been replaced to when fitting a new device becomes possible.

Lockdown initially greatly restricted the provision of LARC methods, with those methods requiring fitting (implant and intrauterine contraception) most affected.

By June 2020 the supply of the implant, IUS and IUD had recovered to only a third of levels in the previous year. Given the potential for LARCs to prevent unwanted pregnancy, and the recent evidence describing the reduction in abortions during the time that GPs were incentivised to supply LARC methods [14], this reduction may lead to increased abortion requests or unwanted pregnancies. However FSRH advice on extending the use of existing intrauterine methods from 5 years to 6 years in the case of 52 mg LNG-IUS and up to 12 years for banded copper IUDs, and of the implant from 3 years to 4 years, may mitigate this risk, provided fitting can return to usual levels in the next 6 months [10].

There was a small increase in the percentage of contraceptive injections supplied sub-cutaneously (SC) compared to IM, suggesting that some women were switching to self-administration, since the SC route is licensed for this.

Prescriptions of emergency contraception dropped sharply in April 20 but quickly rose to pre-lockdown levels by June 2020, which may be due to the ease with which these can be prescribed as part of a telephone consultation.

### Limitations

This paper draws on General Practice prescribing data, so does not reflect the entire range of contraceptive providers. Women can also obtain contraception from community clinics, and emergency contraception from clinics and from community pharmacists. No over the counter hormonal methods are available in the UK, apart from emergency contraception which is available through most pharmacies. It is unlikely that women were prescribed their usual prescription other than from their General Practice since community clinics faced similar restrictions due to COVID-19.

When the association between COVID-19 infection and clotting became known, women and contraceptive prescribers may have been reluctant to use combined hormonal methods, which already carry a known risk of increased clotting. This would present an additional reason for the reduction in combined hormonal prescribing, beyond the need to monitor blood pressure, but it would not account for the reduction in long-acting methods, which are all oestrogen-free.

Throughout this paper it has been assumed that the change in contraceptive prescribing was due to the restrictions on face-to-face consultations and not due to other factors, for which it has not been possible to control. From year to year there is likely to be some fluctuation in contraceptive prescribing and not all of the changes to should be ascribed to the effect of COVID-19. It is a major limitation of this paper that the data for longer sequence of time periods, including a before- and-after COVID-19 series is not yet available and this should be the focus of a future analysis in a few years’ time.

As general practice returns to a more normal form of practice, with face-to-face consultations becoming more easily available, this database should be examined again to see if the change in ratio between COCP and POP persists, in particular if women prescribed the POP as a bridging or ‘stop-gap’ method will return to the COCP. This research has shown that POP prescribing was maintained without face-to-face consultations. The FSRH has called for the Medicines Act to be updated to allow the desogestrel POP to be supplied through pharmacies, without prescription [10]. Cameron et al. have found that women supplied by pharmacists with the POP after a consultation for emergency contraception are more likely to be on an effective contraception in four months later, and Eckhaus et al. in a review have found that both patients and pharmacists believed pharmacy prescribed contraception improved access [15]. Novel guidelines and procedures for supplying contraceptive methods may persist, if found to be beneficial, after the COVID-19 pandemic has ended [16].

## Conclusions

The restriction of access to face-to-face contraceptive consultation in general practice had an effect of contraceptive prescribing and provision during that time.

There was a profound reduction in the provision of LARC methods which may lead to increases in unintended pregnancy and abortion in the next few months.

Prescription and provision of the COCP reduced and provision of the POP remained stable, which is likely to be an effect of the need to monitor blood pressure and BMI for women on the COCP, and the fewer contraindications to the POP. This demonstrates that remote prescription of the POP is feasible, and this enforced change in prescribing habits may inform future guidelines for easing access to the POP without face-to-face consultation with a prescriber.

## Data Availability

Data used in this analysis is publically available at https://www.nhsbsa.nhs.uk/prescription-data/prescribing-data/english-prescribing-data-epd.

## Abbreviations

CHC: Combined Hormonal Contraception
PO: Progestogen Only
COCP: Combined Oral Contraceptive Pill
POP: Progestogen Only Pill
LARC: Long-Acting Reversible Contraceptive method
IUC: Intrauterine Contraception
IUD: Intrauterine Device (copper)
IUS: Intrauterine System (hormonal progestogen only)
SC: Subcutaneous
